# Antifungal Drug Susceptibility of *Candida* Species Isolated from HIV-Positive Patients Recruited at a Public Hospital in São Luís, Maranhão, Brazil

**DOI:** 10.3389/fmicb.2017.00298

**Published:** 2017-03-02

**Authors:** Ana L. G. Terças, Sirlei G. Marques, Eduardo B. Moffa, Márcia B. Alves, Conceição M. P. S. de Azevedo, Walter L. Siqueira, Cristina A. Monteiro

**Affiliations:** ^1^Department Federal Technological Teaching Center of MaranhãoSão Luis, Brazil; ^2^Nucleus of Tropical Pathology and Social Medicine, Department of Pathology, Federal University of MaranhãoSão Luis, Brazil; ^3^Department of Post-Graduate Program in Dentistry, CEUMA UniversitySão Luis, Brazil; ^4^Department of Post-Graduate Program in Parasite Biology, CEUMA UniversitySão Luis, Brazil; ^5^Department of Medicine, Federal University of MaranhãoSão Luis, Brazil; ^6^Schulich Dentistry and Department of Biochemistry, Schulich School of Medicine and Dentistry, The University of Western Ontario, LondonON, Canada

**Keywords:** HIV, AIDS, antifungals, *Candida*, oropharyngeal candidiasis

## Abstract

Oropharyngeal candidiasis is the most common fungal infection in hospitalized patients with acquired immune deficiency syndrome (AIDS). Its progression results in invasive infections, which are a significant cause of morbidity and mortality. This study aimed to quickly and accurately identify *Candida* spp. from oral mucosa of AIDS patients recruited at Presidente Vargas Hospital, in São Luís city, Brazil and to evaluate the sensitivity profile of these fungi to antifungals by using an automated system. Isolates were collected from oropharyngeal mucosa of 52 hospitalized AIDS patients, under anti-viral and antifungal therapies. Patients were included in research if they were HIV-positive, above 18 years of age and after obtaining their written consent. CHROMagar^®^*Candida* and the automated ViteK-2^®^system were used to isolate and identify *Candida* spp., respectively. Antifungal susceptibility testing was performed using the ViteK-2^®^system, complemented with the Etest^®^, using the drugs amphotericin B, fluconazole, flucytosine, and voriconazole. Oropharyngeal candidiasis had a high prevalence in these hospitalized AIDS patients (83%), and the most prevalent species was *Candida albicans* (56%). Antifungal susceptibility test showed that 64.7% of the *Candida* spp. were susceptible, 11.8% were dose-dependent sensitive, and 23.5% were resistant. All the *Candida krusei* and *Candida famata* isolates and two of *Candida glabrata* were resistant to fluconazole. Most of AIDS patients presented oropharyngeal candidiasis and *C. albicans* was the most frequently isolated species. The results showed high variability in resistance among isolated species and indicates the need to identify the *Candida* spp. involved in the infection and the need to test antifungal susceptibility as a guide in drug therapy in patients hospitalized with AIDS. This is the first relate about AIDS patients monitoring in a public hospital in São Luís concerning the precise identification and establishing of antifungal profile of *Candida* spp..

## Introduction

In recent decades, the increasing incidence of fungal infections has been linked to patients with congenital or acquired immunodeficiency (Ortega et al., 2010; Junqueira et al., 2012; Li et al., 2013). *Candida albicans* is the most frequently isolated species in humans (Delgado et al., 2009; Hise et al., 2009; Junqueira et al., 2012; Li et al., 2013). However, there has been a significant increase in the prevalence of infections caused by species of *Candida* other than *C. albicans* such as *C. krusei, C. tropicalis, C. glabrata, C. guilliermondii*, and *C. parapsilosis* (Sant’Ana et al., 2002; Li et al., 2013; Kaur et al., 2016).

Despite the high effectiveness of the current antiretroviral therapies, HIV+ subjects have a higher prevalence of oropharyngeal candidiasis (OPC) than individuals without this disease, and its expression is a predictive marker for increased immunosuppression (Erkose and Erturan, 2007). The advancement of HIV infection can result in more frequent and severe OPC episodes (Sharma et al., 2009). The severity of the disease, associated with debilitating conditions of patients, causes prolonged hospital stays and higher hospital costs, generating a major public health problem (Back-Brito et al., 2009).

The progression of oral candidiasis is often faster and more severe in patients with AIDS due to immunodeficiency and the emergency of antifungal resistance among *Candida* species isolates. Also, in fungal infection, the identification of *Candida* spp. is essential since the pathogenicity profile and sensitivity to a particular antifungal agent vary between different species (Costa et al., 2009; Negri et al., 2009). Some authors also argue that exposure to antifungal agents during candidiasis treatment provided a positive selection pressure for non-*albicans* yeasts, such as *C. glabrata* and *C. krusei* (Hunter et al., 1998; Martinez et al., 2002), that are considered intrinsically less sensitive than others species (Pfaller, 2012).

Therefore, this variability in the behavior of different *Candida* spp. and the increasing number of clinical isolates resistant to current antifungal therapies highlight the need for antifungal susceptibility testing to monitor the antifungal resistance of these microorganisms. This could guide the therapeutic choice and the clinical treatment. In addition, an accurate identification of strains isolated from infections in patients with AIDS is important because these patients are more likely to carry species other than *C. albicans* that may be less sensitive to antifungal agents (Belazi et al., 2005; Li et al., 2013; Idelevich et al., 2014).

Antifungal agents available for the treatment of candidiasis are as follows: the polyenes [nystatin and amphotericin B (AMB)]; the ergosterol biosynthesis inhibitors – the azoles (miconazole, clotrimazole, ketoconazole, itraconazole, and FCZ), allylaminesthiocarbamates, and morpholines; and DNA analog 5-fluorocytosine, and newer agents such as caspofungins (Pappas et al., 2009). The antifungal agents target three cellular components of fungi. Azoles inhibit the synthesis of ergosterol in the endoplasmic reticulum of the fungal cell. Polyenes such as AMB bind to ergosterol in the fungal membrane causing disruption of membrane structure and function. Flucytosine (5-FC) is converted within the fungal cell to 5-fluorouracil, which inhibits DNA synthesis (Patil et al., 2015). All can be used with varying efficacy depending on the type and site of infection and the sensitivity of the *Candida* species (Pfaller et al., 2010). For most *Candida* infections, FCZ is the drug of choice (Pfaller et al., 2010; Patil et al., 2015).

Like many others cities in developing countries such as São Luís in Brazil, antifungal testing is not performed routinely (Hamza et al., 2008; Costa et al., 2009). Also, to the best of our knowledge, there are no studies that have established the prevalence or evaluated the profile of antifungal sensitivity of clinical isolates obtained from AIDS patients in São Luís city, Northeast Brazil. Importantly, the mortality rate for AIDS cases in Maranhão in 2013 is the highest in the Northeast: 6.6 cases per 100,000 inhabitants. In addition, a total of 249 new cases of AIDS has been confirmed in São Luís in 2013 (Brazil, Ministério da Saúde, 2014).

Thus, this study was conducted with the objective of identifying *Candida* spp. from oropharynx in hospitalized AIDS patients using the automated ViteK-2system. We also aimed to evaluate and compare the sensitivity profile of these yeasts to four antifungal drugs, FCZ, AMB, 5-FC, and voriconazole (VCZ), using the ViteK-2 and Etest systems in order to guide the therapeutic choice and the clinical treatment of these patients.

## Materials and Methods

### Patients and Oral Isolates

The *Candida* spp. were obtained by swabbing the oropharyngeal mucosa of 52 AIDS patients hospitalized in Getúlio Vargas Hospital in São Luis, Brazil. Patients were included in research if they were HIV-positive, above 18 years of age and submitted their written consent (Ethics Committee of Federal University of Maranhão process No. 23115006540/2009-40). Some patients were under anti-viral and antifungal therapies. Among the patients, there were 22 females and 30 males in the age range of 19–61 years.

The patients in this study presented CD4+ cell counts ranging from 10 to 552 cells/mm^3^ and viral loads of 2,567 to 256,860 copies/mL. Sterile swabs were used for the collection of samples and were inoculated into tubes containing saline solution P.A 0.85% (ISOFAR, Paraná, Brazil). They were then sent to the Medical Mycology Laboratory of Ceuma University and were incubated in BHI (*Broth Heart Infusion* – Acumedia Manufactures) at 37°C for 48 h.

### *Candida* spp. Identification and Preparation of Inoculums

The primary isolation of yeasts was performed using the CHROMagar^®^*Candida* (Difco) Chromogenic differential medium in Petri dishes and incubated at 37°C for 48 h. This medium is based on the use of β-glucosaminidase substrate and differentiates yeast according to the morphology and the color of the colonies. This method provides a presumptive diagnosis of *Candida* spp. (Hospenthal et al., 2004). Green colored colonies were identified as *C. albicans*, blue-cobalt as *C. tropicalis*, pink or lilac as *C. krusei*, and other species were whitish-pink in color. The isolated yeasts from chromogenic medium were picked and incubated at 35°C for 48 h in tubes with SDA (*Sabouraud Dextrose Agar with chloramphenicol* – Acumedia) medium and then stored at -20°C for use in the study.

The biochemical identification of the yeasts was performed using an automated method (Vitek-2 Compact bioMérieux, Marcy-l’Étoile, France).

The Biomerieux Vitek-2 system includes the Vitek-2 cards that allow species identification by comparison of the biochemical profile with an extensive database http://www.jgid.org/article.asp?issn=0974-174777X;year=2016;volume=8;issue=4;spage=139;epage=146;aulast=Kaur-ref9 (Cuenca-Estrella et al., 2010). Biomerieux Vitek-2 expanded its role in this area with a yeast susceptibility test that determines *Candida* growth spectrophotometrically using Vitek-2 microbiology systems, performing fully automated testing of susceptibility to 5-FC, AMB, FCZ, and VCZ (Borghi et al., 2010).

For the preparation of the fungal inoculum (3 mL 0.45% saline + yeast colony), a McFarland scale 2 from DensiChek-bioMerieuxVitek^®^ system was used. This standardized suspension was aspirated into the identification cards, and then the cards were sealed and subjected to biochemical tests by an optical sensor reading. We used the YST card (*Yeast identification*, bioMérieux) to determine the genus and species of yeast. The test was considered complete when the percentage of probability was ≥85% and there was no request for further testing.

#### *In vitro* Antifungals Susceptibility Tests

Thereafter, antifungal susceptibility tests (AFST) were conducted using the ViteK-2^®^ automated system and Etest^®^ (Biodisk AB, Solna, Sweden) according to the manufacturer’s recommendations. These methods have been chosen because they are easy to perform and offer results in a short period of time (Kaur et al., 2016).

For this, 180 μL of inoculum was standardized to the McFarland scale 2.0 using the DensiChek densitometer of the ViteK-2^®^ system, placed in a tube containing 3 mL of 0.45% saline, and aspirated into the AST-YSO1 card (bioMérieux). The following antifungal drugs were tested: AMB (0.03–16 μg/mL), FCZ (1–64 μg/mL), 5-FC (0.125–64 μg/mL), and VCZ (0.125–16 μg/ml). The analysis and interpretation of data were performed according to M27-A3 and M27-S3 CLSI standards. For quality control, we used standard strains of *C. krusei* (ATCC 6258) and *C. parapsilosis* (ATCC 22019). For fungal strains that did not respond to the AST cards, an Etest^®^ (Biodisk AB, Solna, Sweden) was performed, which consisted of a gradient method with predefined concentrations of FCZ and AMB in μg/mL. In this method, the colonies were seeded at a concentration of 0.5 McFarland on dishes with RPMI (Probac^®^, Sao Paulo, Brazil) supplemented with 2% glucose agar and then incubated at 35°C for 48 h. The reading was performed by assessing the point of intersection between the halo formed and the Etest strip. The FCZ MIC was determined as the lowest concentration that inhibited 80% of fungal strains, and the AMB MIC was calculated as the lowest concentration with no observed fungal growth. The profile of the antifungal drug sensitivity was classified as sensitive (S), dose-dependent sensitivity (S-DD), and/or intermediate (I), and resistant (R).

The breakpoints used to define sensitivity, intermediate, and resistant for each species were those defined by Clinical and Laboratory Standards Institute [CLSI] (2008). The MIC values ≤ 8 μg/mL for FCZ were considered susceptible (S), 16–32 μg/mL was considered as susceptible dose-dependent (SDD), and ≥64 μg/mL as resistant (R). For AMB, MICs ≤ 1 μg/mL were considered to be S and ≥1 μg/mL was R. For 5-FC, CIMs ≤ 4 μg/mL were considered to be S, 8–16 μg/mL was I, and ≥32 μg/mL was R. For VCZ, MICs ≤ 0,125 μg/mL were S and ≥16 μg/mL were considered R.

Fluconazole and amphotericin were chosen for the tests because they have different mechanisms of action and are the main drugs chosen for the treatment of *Candida* infections (Patil et al., 2015). VCZ and 5-FC were chosen because they could be an alternative for species resistant to FCZ and amphotericin.

### Ethics Statement

The study protocol was established according to the Guidelines and Standards for Research Involving Humans (Resolution of the National Council No. 196/96 of October 10, 1996) and was approved by the Ethics Committee of UFMA (Federal University of Maranhão) under decision No. 23115006540/2009-40. All the participants signed a free and informed consent form.

### Statistical Analysis

The data collected were expressed as mean and standard deviation for numeric variables, and absolute and relative frequencies for categorical variables. We used the chi-squared (χ^2^) test to analyze categorical variables, and analysis of variance (ANOVA) followed by the Tukey’s post-test was used for numeric variables when *p* < 0.05. A significance level of *p* < 0.05 was adopted.

## Results

### Patient Data and Prevalence of Isolates

From 52 AIDS patients participating in the study, 43 were positive for *Candida.* Fifty-four fungal isolates were recovered from these patients, and thus, in some cases, more than one species was isolated from a single clinical sample. Eighty-three percent of patients participating in the study had oropharyngeal candidiasis, and 52.2% had used antifungals. Of these, 75% were using both FCZ and nystatin and 8.3% used FCZ, nystatin, and AMB.

Among the yeast samples, 43 (83%) grew in CHROMagar Candida medium and 9 (17%) were negative. Using the automated Vitek-2^®^ Compact bioMérieux system, 51 isolates were identified as *Candida* spp., including 29 *C. albicans* (56%), 6 *C. tropicalis* (12%), 6 *C. krusei* (12%), 4 *C. glabrata* (8%), 2 *C. famata* (4%), 2 *C. parapsilosis* (4%), and 2 *C. guilliermondii* (4%) (**Table 1**). One isolate of *Kodamaeaohmeri* (*Pichia ohmeri*), one of *Rhodotorula* spp., and one of *Trichosporon* spp. were also identified.

**Table 1:** Number and prevalence (%) of the *Candida* strains identified by the Vitek-2^®^ system, and their distribution according to patient gender.

Species	N. Isolates	Female	Male
*Candida albicans*	29(56%)	11	18
*Candida tropicalis*	6 (12%)	3	3
*Candida krusei*	6(12%)	2	4
*Candida glabrata*	4 (8%)	2	2
*Candida guilliermondii*	2 (4%)	1	1
*Candida parapsilosis*	2 (4%)	1	1
*Candida famata*	2 (4%)	2	0
Total	51	22 (43%)	29 (57%)

*Chi-squared test (χ^2^): there was no statistically significant difference in the incidence of *Candida* spp. analyzed according to gender (*p* = 0.74).*

**FIGURE 1:**
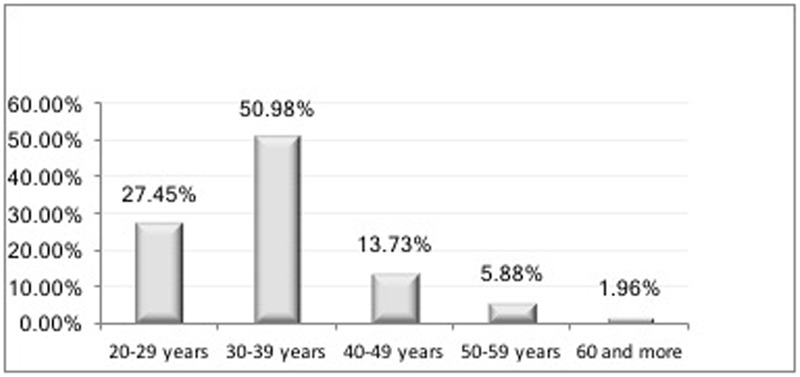
**Distribution (%) of hospitalized patients with acquired immune deficiency syndrome (AIDS) by age group (years)**.

When looking at the distribution of isolates of *Candida* spp. by patients’ gender, we observed that the number of isolates coming from the men patients (*n* = 29/57%) was higher than that from the women (*n* = 22/43%; **Table 1**). The age group with the highest frequency of *Candida* spp. isolates was 30–40 years (50.98%), followed by the 20–29 years age group (27.45%; **Figure 1**).

### *In vitro* Susceptibility of Isolates

The antifungal susceptibility testing performed by the automated ViteK-2^®^ system showed that 58% of tests were completed in a period of 12–15 h, and most of them did not exceed 19 h. However, for four slow-growing or nutritionally fastidious and demanding microorganisms, 36 h was not sufficient to obtain the results. These cases included the following isolates: 2 *C. famata*, 1 *Rhodotorulaglutinis*, 1 *Trichosporon* spp., and 1 *Kodamaeaohmeri* (*Pichia ohmeri*). For these isolates, the MIC was determined using the Etest^®^ system. The results showed that all strains of *C. albicans* (*n* = 29), *C. tropicalis* (*n* = 6), *C. parapsilosis* (*n* = 2), and *C. guilliermondii* (*n* = 2) showed sensitivity to all antifungals drugs tested. All six isolates of *C. krusei* were resistant to FCZ, four of them showed intermediate susceptibility to 5-FC, and all were sensitive to AMB and VCZ (**Table 2**). Two *C. glabrata* isolates were resistant to FCZ, one was resistant to AMB, and another one was resistant to VCZ. The sensitivity profile of the antifungals tested was virtually the same for all of the *Candida* spp. used in the survey, except for *C. krusei* and *C. famata*, which were resistant to FCZ. *C. glabrata* isolates showed resistance to three different antifungals. However, this small difference in sensitivity toward the antifungals in these three species did not translate to a statistically significant difference. The ANOVA test showed no difference in the sensitivity profile (*p* = 0.99), S-DD (*p* = 0.51), and resistance (*p* = 0.18) of the antifungals.

**Table 2:** Distribution of isolates of *Candida* spp. according to the minimum inhibitory concentration (MIC) against four antifungal drugs as evaluated by the Vitek 2^®^ system.

Species *Candida* spp.	No. (%) Sensitivity Profile			
	Total (%)	*S* (%)	S-DD(I) (%)	*R* (%)
***C. albicans***	**29 (56)**			
Fluconazole		29 (100)	0	0
Amphotericin B		29 (100)	0	0
Voriconazole		29 (100)	0	0
Flucytosine		29 (100)	0	0
***C. tropicalis***	**6 (12)**			
Fluconazole		6 (100)	0	0
Amphotericin B		6 (100)	0	0
Voriconazole		6 (100)	0	0
Flucytosine		6 (100)	0	0
***C. glabrata***	**4 (8)**			
Fluconazole		2 (50)	0	2 (50)
Amphotericin B		3 (75)	0	1 (25)
Voriconazole		3 (75)	0	1 (25)
Flucytosine		4 (100)	0	0
***C. krusei***	**6 (12)**			
Fluconazole		0	0	6 (100)
Amphotericin B		6 (100)	0	0
Voriconazole		6 (100)	0	0
Flucytosine		2 (33)	4(67)	0
***C. guilliermondii***	**2 (4)**			
Fluconazole		2 (100)	0	0
Amphotericin B		2 (100)	0	0
Voriconazole		2 (100)	0	0
Flucytosine		2 (100)	0	0
***C. parapsilosis***	**2 (4)**			
Fluconazole		2 (100)	0	0
Amphotericin B		2 (100)	0	0
Voriconazole		2 (100)	0	0
Flucytosine		2 (100)	0	0

*Classification according to the rules of the Clinical and Laboratory Standards Institute M27-A3: **Fluconazole** (*S* ≤ 8 μg/mL; S-DD, 16–32 μg/mL; *R* ≥ 64 μg/ml); **Voriconazole** (*S* ≤ 1 μg/mL; SDD, 2 μg/mL; *R* ≥ 4 μg/mL); **Flucytosine** (*S* ≤ 4 μg/mL; I, 8–16 μg/mL; *R* ≥ 32 μg/mL); **Amphotericin B** (*S* < 1 μg/mL; *R* > 2 μg/mL). S, sensitive; S-DD, dose-dependent sensitivity; I, intermediate sensitivity; R, resistant.*

In the susceptibility test performed using the Etest^®^ method, two isolates of *C. famata* were resistant to FCZ but were susceptible to AMB. *Rhodotorulaglutinis* isolates were resistant to FCZ and AMB. *Trichosporon* spp. isolates were susceptible to FCZ and resistant to AMB. *Kodamaeaohmeri* (*Pichia ohmeri*) was resistant to FCZ but it displayed sensitivity to AMB (**Table 3**).

**Table 3:** Minimum inhibitory concentrations (MIC) as evaluated by the Etest^®^system of four different fungals.

Fungal species	Fluconazole	Amphotericin B
*Candida famata*	128 (R)	0.75 (S)
*Kodamaeaohmeri*	>256 (R)	0.38 (S)
*Rhodotorulaglutinis*	>256 (R)	2 (R)
*Trichosporon* spp.	3 (S)	32 (R)

*Classification according to the rules of the Clinical and Laboratory Standards Institute M27-A3: **Fluconazole** (*S* ≤ 8 μg/mL; S-DD, 16–32 μg/mL; *R* ≥ 64 μg/mL); **Amphotericin B (***S* < 1 μg/mL; *R* > 1 μg/mL). S, sensitive; S-DD, dose-dependent sensitivity; R, resistant.*

## Discussion

Oropharyngeal candidiasis is the most common opportunistic infection among HIV-seropositive patients and in those with AIDS, and it represents a major treatment challenge. Hence, it is recommended to determine the species involved in the infection and its antifungal susceptibility.

Among 52 patients involved in this study, 30 were men and 22 were women, resulting in the ratio of 1.4 cases in men for every 1 case in women. These findings lead us to reflect on whether the incidence of AIDS in women is increasing and equating with the incidence in males. Data from the last epidemiological study in Brazil, published in the AIDS and STDs Bulletin in 2011, showed that in 2011, the ratio of 1.7 cases in men for every 1 case in women was greatly diminished when compared to data from 1989, when there were about six cases of AIDS in men for every case in women (Hinrichsen et al., 2008). In the present study, we also found that the most prevalent age group was 30–39 years, followed by the 40–49 years age group. These data are similar to those published in Brazil (Hinrichsen et al., 2008) and other countries (Hamza et al., 2008).

The patients hospitalized with AIDS in this study presented a low CD4+ lymphocyte count (90% of patients had less than 200 cells/mm^3^) and a high viral load (80% of patients had above 50,000 copies/mL). Although 90% of patients had CD4+ lymphocyte counts below 200 cells/mm^3^, studies have not shown any correlation between oral candidiasis and low CD4+ lymphocyte counts, but they have shown correlation with a high viral load (Morgan, 2005; Erkose and Erturan, 2007; Back-Brito et al., 2009). This study also showed a high frequency of OPC in these patients (83%), and most of them had exposure to more than one antifungal agent, including FCZ, nystatin, and AMB. These data are alarming and support the relevance of our research since we isolated a very high frequency of resistant yeasts. As far as we know, this is the first study with AIDS patients from the city of São Luís concerning the distribution of oral yeasts, species prevalence, and antifungal susceptibility profile.

The isolation of *Candida* species in this study was performed by the CHROMagar *Candida* culture medium, and consequently, it presumptively identified some species involved. Thereafter, we used the ViteK-2system, a system with excellent reproducibility and accuracy when compared with the CLSI method (Clinical and Laboratory Standards Institute [CLSI], 2008; Bourgeois et al., 2010; Cuenca-Estrella et al., 2010; Kaur et al., 2016) for identification of all isolates. Among the 52 patients admitted with AIDS who submitted to buccal smears, 43 (83%) were positive for *Candida* ssp. culture. This result is similar to that found in other studies, which showed that 80–95% of these patients develop one or more fungal infections during their illness (Rex et al., 2000; Campisi et al., 2002, Junqueira et al., 2012). In this study, we observed that *C. albicans* was the most prevalent species (56%) against all non-*albicans Candida* species (NAC; 44%), which is in accordance with other studies (Viudes et al., 2002; Colombo et al., 2006; Kaur et al., 2016). Among the NAC species, the most prevalent species were *C. tropicalis, C. krusei*, and *C. glabrata*, which is in agreement with other studies (Colombo et al., 2006; Hamza et al., 2008). In this study, we identified a case of a patient with four *Candida* spp. that were identified in a single clinical sample; one of the species was *C. glabrata*, which is resistant to both FCZ and AMB, and the other was *C. krusei*, which is resistant to FCZ. This finding is relevant because FCZ is the drug of choice for candidiasis treatment in AIDS patients although it has a fungistatic action (Siikala et al., 2010; Rautemaa and Ramage, 2011), and both FCZ and AMB were being used by some of the patients who participated in the research. Seven patients had double colonization, and one of them had colonization by *C. krusei* resistant to FCZ. The coexistence of various species in the same clinical specimen has also been reported in other studies (Swinne et al., 2004; Junqueira et al., 2012).

We used the automated ViteK-2^®^ and Etest^®^ systems to screen for AFST because they present good reproducibility and rapid diagnostic tests with *Candida* spp. (Pfaller et al., 2007; Borghi et al., 2010; Bourgeois et al., 2010; Cuenca-Estrella et al., 2010; Kaur et al., 2016). Both methods have advantages over the Clinical and Laboratory Standards Institute (CLSI) standardized broth microdilution method, which is considered as a reference for antifungal susceptibility testing although it is complex and laborious to use as a routine method (Bourgeois et al., 2010; Kaur et al., 2016). The VK2 method demonstrated excellent reproducibility, which underscores its excellent level of standardization. Spectrophotometric readings remove subjectivity from the MIC determination. Furthermore, *Candida* species identification and *in vitr*o antifungal susceptibility are obtained in less than 26.5 h (mean, 15.2 h), thus, reducing the time necessary for optimizing antifungal treatment decisions. ViteK-2^®^ system results are on average available within 15 h 13 min, with a range of 12 h 15 min (*C. albicans*) to 26 h 30 min (*C. glabrata*; Bourgeois et al., 2010). The Etest^®^ method is an alternative, standardized, and reliable method adapted to hospital laboratories. This method provides an accurate MIC value, and this critical information helps the clinician to ensure that the appropriate treatment of the patient will be achieved (Pfaller et al., 1998; Bourgeois et al., 2010).

Nevertheless, in this study, some fastidious species failed to grow sufficiently when we used the automated ViteK-2^®^ system, but for the Etest^®^ method, all of the isolates grew enough to be read at 24 and 48 h. Thus, we note that, when used in conjunction with the ViteK-2^®^ method, these two AST methods have the potential to satisfy all the requirements for susceptibility testing in routine clinical microbiology analysis. Generally, many authors use one or another method, mainly the classical, laborious, and time consuming microdilution method described in the CLSI M27-A3 standard (Bremenkamp et al., 2011; Melo et al., 2011; Li et al., 2013; Sanitá et al., 2013), which is still considered the gold standard for susceptibility tests.

In this study, all isolates of *C. albicans* (*n* = 29), *C. tropicalis* (*n* = 6), *C. parapsilosis* (*n* = 2), and *C. guilliermondii* (*n* = 2) showed sensitivity to all of the antifungal drugs tested, which is consistent with the general pattern of susceptibility of the NCLS M-27 method and with the results of other studies (Pfaller et al., 2007, 2008). Eighty percent of the *Candida* spp. studied were sensitive to FCZ, 18% were resistant, and 2% presented a S-DD. These results are similar to those observed by other researchers (Hospenthal et al., 2004; Pfaller et al., 2007; Hamza et al., 2008). Resistance to FCZ was observed in this study in all six samples of *C. krusei* (100%), two of *C. glabrata* (50%), and all of *C. famata* (100%). In fact, *C. krusei* has an innate resistance to FCZ, and *C. glabrata* possesses the capacity to develop resistance after the first contact with this antifungal agent. The results of this study are in agreement with several studies that demonstrated the innate resistance of *C. krusei* to FCZ and the increased resistance of C. *glabrata* and C. *famata* species to this antifungal drug (Campisi et al., 2002; Hamza et al., 2008; Pfaller et al., 2008; Junqueira et al., 2012; Patil et al., 2015).

Regarding VCZ, almost all *Candida* spp. isolates in this study were sensitive to this drug, and only *C. glabrata* demonstrated resistance. Based on these data, VCZ could be an effective drug for the treatment of patients involved in the study. In fact, studies have shown that VCZ is effective against *Candida* spp. that were resistant to FCZ and/or AMB (Swinne et al., 2004; Pfaller et al., 2007).

For AMB, the cutoff point (breakpoints) had not been determined by CLSI. Researchers have determined that a value of <1 μg/mL for an isolated strain would show sensitivity to AMB, and a value of >1 μg/mL would demonstrate resistance (Hepburn et al., 2003; Kaur et al., 2016). According to these cutoffs, this study showed that 94% of the *Candida* isolates were sensitive, 2% presented S-DD, and 4% were resistant. These results were similar to those from other studies, where upon examination of 100 HIV-infected patients revealed 58 with positive cultures for *Candida* spp., and of these, 96% were susceptible to AMB and 4% were resistant (Alves et al., 2006). Despite 50 years of polyene use, resistance to AMB is rare because it binds to ergosterol in the fungal membrane causing disruption of membrane structure and function, having, thus, a fungicide action (Patil et al., 2015). However, it is worth noting that among the non-*Candida* yeasts isolated in this study, two were resistant to AMB and two were resistant to FCZ. One of them was resistant to both antifungals, which draws our attention to the danger of treatment without the precise identification and establishment of the antifungal profile.

With regard to 5-FC, a high sensitivity of *Candida* isolates (92%) was observed in this study probably because this antifungal inhibits DNA synthesis (Patil et al., 2015). Only four (8%) *C. krusei* samples showed S-DD. Flucytosine has a high activity against *C. albicans*, although it has been reported that 40% of *C. krusei* strains are resistant to this drug. Flucytosine should be used as a treatment option only when the *Candida* spp. is resistant to the azole antifungal family (Alves et al., 2006).

Many public hospitals that treat patients with HIV/AIDS do not have the laboratory facilities that allow a rapid and accurate diagnosis for treatment planning. Our results showed that OPC infection has a high prevalence in hospitalized AIDS patients (83%), and although all *C. albicans* were sensitive to the drugs tested, most of the NAC species showed high resistance to more than one antifungal agents (30%). Most of the observed resistance was to FCZ, and the species that highlights as resistant were *C. krusei* (100%), *C. famata* (100%), and *C. glabrata* (50%). Also, 25% of *C. glabrata* were resistant to AMB and 40% of *C. krusei* were intermediate to 5-FC. These findings are important because they show the variable resistance profile among different strains and the need to identify the *Candida* spp. involved in the infection, mainly because the patients involved were or had been on antifungal treatment. This also demonstrates the use of antifungal susceptibility testing as a guide to drug therapy in hospitalized AIDS patients.

Determining the MIC of a drug does not guarantee the success of the treatment because, in an infection, the role of the host is critical to obtain a satisfactory clinical response. However, it is known that when the profile of a *Candida* spp. is resistant at a tested drug concentration, this concentration often leads to a failed clinical treatment (Rex et al., 2000).

We found a high prevalence of OPC and high frequency of resistant *Candida* spp. in AIDS patients in a public hospital in Sao Luis. São Luís is a city in the northeast of Brazil with the highest number of cases of AIDS, and like other cities in developing countries, *in vitro* antifungal testing is not performed routinely. Monitoring clinical samples is extremely important for the selection of the appropriate drug and dose, which helps predict those patients who are likely to respond to therapy and ensure prevention of excessive dosing and selection of resistant microorganisms. The data obtained here allowed the most effective monitoring of these patients, providing them with the possibility of more adequate therapy. It is noteworthy that the Vitek-2^®^ automated system used in conjunction with the Etest^®^ meets all the requirements for the susceptibility testing of fungi in routine clinical microbiology laboratories.

## Author Contributions

Conceived and designed the experiments: AT, SM, CdA, MA, and CM. Performed the experiments: AT, SM, CdA, and CM. Analyzed the data: AT, SM, EM, MA, WS, and CM. Contributed reagents/materials/analysis tools: AT, SM, EM, MA; CdA, WS, and CM. Wrote the manuscript: AT, SM, EM, MA, CdA, WS, and CAM.

## Conflict of Interest Statement

The authors declare that the research was conducted in the absence of any commercial or financial relationships that could be construed as a potential conflict of interest.
